# Epigenetic Landscapes of Methamphetamine Addiction: Unravelling the Diagnostic Potential of Gene Methylation

**DOI:** 10.1111/adb.70108

**Published:** 2025-12-10

**Authors:** Qingxiao Hong, Shanshan Chen, Zhongze Lou, Weisheng Chen, Han Du, Longhui Li, Xiaohu Xie, Wenjin Xu, Wenhua Zhou, Huifen Liu

**Affiliations:** ^1^ Department of Psychiatry Affiliated Kangning Hospital of Ningbo University, Ningbo University Ningbo Zhejiang China; ^2^ Zhejiang Key Laboratory of Drug Addiction and Brain Health Ningbo Kangning Hospital Ningbo Zhejiang China; ^3^ Yuyao Third People's Hospital Ningbo Zhejiang China; ^4^ Department of Psychosomatic Medicine The First Affiliated Hospital of Ningbo University Ningbo Zhejiang China

**Keywords:** addiction, ATP6V1C1, CES1, GABRB1, KCNQ2, methamphetamine, methylation, USP7

## Abstract

Methamphetamine addiction is a chronic brain disorder involving significant neuroadaptive changes, with recent research emphasizing the role of epigenetic mechanisms, particularly DNA methylation. This study aims to evaluate the diagnostic potential of gene methylation by identifying and validating differentially methylated genes in methamphetamine‐dependent individuals versus healthy controls. A genome‐wide differentially methylated analysis was conducted using methylation microarray technology. Subsequently, pyrosequencing was employed for validation with an expanded sample size, examining 27 CG sites across eight candidate genes: ATP6V1C1, CES1, USP7, GABRB1, KCNQ2, LIAS, CIZ1 and GNG7. ROC curve analyses and correlation assessments with biochemical markers and drug use patterns were also performed. Significant methylation alterations were observed in GABRB1, CES1, KCNQ2 and USP7 between methamphetamine‐dependent individuals and controls. Specifically, GABRB1 and KCNQ2 showed decreased methylation, while CES1 exhibited increased methylation. USP7 displayed site‐specific changes. ROC curve analysis showed that a specific site in the GABRB1 gene demonstrated excellent diagnostic accuracy (AUC = 0.902). Methylation levels in CG sites in CES1 showed high diagnostic accuracy (AUC = 0.755) for methamphetamine dependence, while the AUC values for KCNQ2 and USP7 were 0.68 and 0.664, respectively, indicating moderate classification. Besides, the study revealed significant positive correlations between diastolic pressure and both the duration of methamphetamine use and KCNQ2 methylation levels. Additionally, USP7 methylation levels showed a positive correlation with the duration of drug use. These findings provide valuable insights for the development of diagnostic biomarkers and targeted therapeutic interventions. Future research will focus on elucidating the functional roles of these genes in the pathophysiology of methamphetamine addiction and their potential applications in treatment strategies.

## Graphical abstract

This study identified genome‐wide DNA methylation alterations in methamphetamine‐dependent individuals. Validation confirmed significant methylation changes in GABRB1, CES1, KCNQ2, and USP7, some of which correlated with drug use history. Notably, GABRB1 methylation demonstrated excellent diagnostic accuracy (AUC=0.902), highlighting its potential as a novel epigenetic biomarker for methamphetamine addiction diagnosis and treatment targeting.

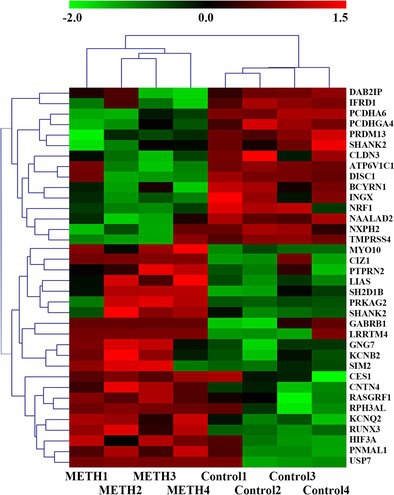

## 1 Introduction

Methamphetamine is a prevalent and highly addictive psychostimulant [1]. Continuous consumption of methamphetamine results in drug dependency, a persistent and recurring condition marked by uncontrollable use and strong desires for the substance [2]. The hallmark of methamphetamine addiction lies in a repetitive cycle encompassing drug intake, withdrawal symptoms and relapse [3]. Consistent use of methamphetamine over an extended period can elicit a range of psychopathology‐mimicking behaviours, encompassing hallucinations, paranoia, aggressive tendencies, suicidal ideation and emotional disturbances [4, 5, 6, 7]. Conversely, sudden discontinuation can trigger fatigue, depressive states and aggressive outbursts [8], posing profound health risks and societal concerns. The China Drug Situation Report 2023 shows that methamphetamine abuse accounts for 50.8% of total drug abuse cases, with approximately 455 000 individuals affected. Methamphetamine addiction represents a significant public health issue, affecting millions of individuals worldwide, highlighting the urgent need for effective intervention and therapeutic strategies.

Growing body of evidence highlights the complex interplay between DNA methylation, gene expression regulation and neuroplasticity in methamphetamine addiction, providing crucial insights for developing more effective treatment strategies for substance use disorders. For example, subchronic methamphetamine treatment induces distinct patterns of DNA methyltransferases (Dnmt1) mRNA expression in the caudate nucleus and nucleus accumbens of two inbred rat strains [9, 10]. Methamphetamine dependency was shown to be associated with reduced DNA methylation and a corresponding increase in the expression of several key genes involved in the pathogenesis of psychotic disorders, such as DRD3, DRD4, MB‐COMT and AKT1 genes [11]. Repeated methamphetamine abuse is associated with DNA methylation at specific CpG sites including CNOT1 and PUM1 genes [12]. A previous study also showed a significant correlation exists between BDNF methylation and addiction phenotypes, including tension‐anxiety, anger‐hostility, fatigue‐inertia and depression‐dejection [13]. Increased CpG methylation in the promoter of GRM8 is a potential candidate epigenetic biomarker of psychotic symptoms in methamphetamine‐induced psychotic disorder [14].

Despite these advances, significant knowledge gaps persist in understanding the specific epigenetic signatures associated with methamphetamine addiction. This research seeks to expand current evidence by identifying methylation differences across the genome using the Illumina methylation array and validating candidate genes through pyrosequencing, ultimately evaluating their diagnostic potential through receiver operating characteristic (ROC) curve analysis. Additionally, we will explore correlations between biochemical markers and patterns of drug use to further characterize the epigenetic landscape of addiction.

## 2 Methods

### 2.1 Sample Collection

Fifty men from Ningbo Compulsory Isolation Drug Rehabilitation Centre and Ningbo Addiction Research and Treatment Center were recruited for the present study; they all met the Diagnostic and Statistical Manual of Mental Disorders (Fifth Edition) criteria for methamphetamine dependence. Forty healthy participants were recruited as the healthy controls from Ningbo Health Screening Centre. The mean age of methamphetamine‐dependent individuals was 35.12 ± 7.41 years; healthy normal controls were 36.20 ± 10.87 years. For the Illumina methylation array study, a discovery subset of eight participants—four methamphetamine‐dependent cases (mean age: 35.50 ± 1.66 years) and four healthy controls (mean age: 35.75 ± 2.28 years) was selected from this total cohort of 90 individuals. All participants were male. Peripheral blood samples were collected; all of them participated in the study voluntarily and provided written informed consent. The study was reviewed and approved by Ningbo Addiction Research and Treatment Center (Ethical Review No. 201701, China).

### 2.2 Methylation Array Testing and Analysis Process

DNA extraction was performed using the DNeasy Blood & Tissue Kit (QIAGEN) following the manufacturer's instructions. Bisulfite conversion of unmethylated cytosines to uracils was carried out using the EZ DNA Methylation Gold Kit (Zymo Research). Quality control was ensured by using synthetic templates to evaluate experimental performance.

Illumina iScan system was used to detect methylation patterns using the Infinium Methylation EPIC Bead Chip. This involved DNA amplification, fragmentation, hybridization to the beadchip and scanning with the iScan system to capture high‐resolution images for precise methylation quantification at over 850 000 CpG sites per sample. Raw data were processed in GenomeStudio software, yielding methylation scores (*β* values) for each CpG site. Initial quality control excluded samples with detection *p* values > 0.01 for > 1% of probes. Background noise was corrected using built‐in negative controls, and data normalization was applied using internal controls and quantile normalization. Bisulfite conversion efficiency was verified using control probes, and batch effects were corrected with the ComBat algorithm.

### 2.3 Differential Methylation Analysis

Methylation status at each CpG site was determined by calculating *β* values (methylated/total probe intensity). For genome‐wide methylation difference analysis, *β* values were used as input in the limma R package. Linear modelling and empirical Bayes moderation were applied to compare methylation levels between methamphetamine‐dependent individuals and healthy controls. Given that this stage was designed as an exploratory pilot study with a limited sample size (*n* = 4 per group), we used uncorrected (nominal) *p* values to rank and select the top candidate CpG sites for subsequent validation. This approach balanced the risk of false negatives (Type II errors) and false positives (Type I errors) at the screening stage, ensuring that potentially important biological signals were not prematurely discarded.

### 2.4 Identification of Significantly Differentially Methylated Positions (DMPs)

Significant DMPs were identified based on an *p* value threshold of less than 0.05 and an absolute delta *β* values (difference in methylation percentage) of at least 5%. These criteria ensured that only the most biologically relevant methylation changes were considered for further analysis. Data on methylation levels of differentially methylated sites were entered using the Complex Heatmap package in R, normalizing the data for easy comparison.

### 2.5 Gene Ontology (GO) and Kyoto Encyclopedia of Genes and Genomes (KEGG) Analysis

Genes that are associated with the identified DMPs were subjected to GO and KEGG pathway enrichment analyses to understand the biological processes and pathways potentially involved in methamphetamine addiction, which provides insights into the functional implications of the observed methylation changes. Bioinformatic analysis and enrichment scatter plot were performed using the OmicStudio tools available at https://www.omicstudio.cn/tool. In the enrichment analysis, GO terms and KEGG pathways with a *p* value < 0.05 were considered statistically significant. For visualization in the enrichment scatter plot, the top 10 most significant terms or pathways ranked by raw *p* value were selected.

### 2.6 Heatmap Visualization

Heatmaps were generated using the pheatmap R package to display the methylation patterns of the top differentially methylated genes (DMGs) across all samples. The appropriate colour mapping was set to show hypermethylated and hypomethylated sites clearly, providing an intuitive overview of the data structure and highlighting the methylation differences between the addicted and control groups. The ggplot2 package in R was used; volcano plots were plotted to visualize the statistical significance and effect size of differentially methylated loci. To prioritize candidate genes with relevance to addiction, the protein‐coding genes associated with the identified DMPs were cross‐referenced with the Knowledge of Addiction Related Genes‐Human (KARG) database. The complete list of 1670 genes obtained from the KARG database is provided in Supporting Information S2 for transparency and reproducibility.

### 2.7 Pyrosequencing Validation of Candidate Genes

#### 2.7.1 Experimental Design for Pyrosequencing

Pyrosequencing was utilized to validate the DMGs. A significant difference (*p* value < 0.05) and an absolute methylation difference |*β* difference| > 0.14 were used as the screening criteria for differentially methylated loci between groups, and the test results were subsequently analysed. Then, based on the results of the premethylation microarray data, we screened eight of the most interesting genes, which exhibited the highest delta *β* values and were biologically relevant to addiction pathways observed in the array data.

The PCR amplification of bisulfite‐treated DNA was followed by sequencing on the PyroMark Q48 System. Primers for the pyrosequencing assay were designed using the PyroMark Assay Design software. The information of the primers is shown below (Table 1), including ATPase H + transporter V1 subunit C1 (ATP6V1C1), carboxylesterase 1 (CES1), ubiquitin‐specific peptidase 7 (USP7), ribosomal protein L9 (RPL9)/lipoic acid synthetase (LIAS), γ‐aminobutyric acid (GABA) A‐type receptor β1 subunit (GABRB1), potassium voltage‐gated channel KQT‐like subfamily member 2 (KCNQ2), CDKN1A interacting zinc finger protein 1 (CIZ1), and guanine nucleotide binding protein (G protein) γ7 gene (GNG7).

**TABLE 1:** Detailed information on the differential methylation sites and pyrosequencing primer sequences.

Gene	Sites	Primer	Sequence (5′‐3′)	*p* *	*β* difference	Mean‐meth	Mean‐control
ATP6V1C1	cg14877637	Forward	GTGGTAAGGAGTGATTTTTTTTAGTAG	0.0312	−0.1490	0.6273	0.7763
		Reverse	Biotin‐CCAACCCCCTACAAAAACTTC				
	cg22150661	Sequencing	AGTGATTTTTTTTAGTAGGTAA	0.0399	−0.1425	0.4714	0.6138
CES1	cg23196985	Forward	Biotin‐AAGTAGAGAGAGTGGTTAGGATAAAG	0.0455	0.1880	0.6698	0.4818
		Reverse	AAAAACTTTTCTAATCTCTCCCAATTAA				
		Sequencing	CTAAACTACACAAAAACCT				
USP7	cg01891583	Forward	TAGTTGATTTAGAGTTGGTTGTTAGTGG	0.0202	0.3879	0.5456	0.1577
		Reverse	Biotin‐TTCTACAAAAAAAAAAAACTTAACCCTATA				
		Sequencing	GATTTTATTTAAGTTTGATAAAGA				
LIAS	cg19311470	Forward	TTTTAGAGTAGATGGTTTTAGATTTTTAGT	0.0416	0.4908	0.8068	0.3160
		Reverse	Biotin‐CAAACTACTCCACCATTAACTACTTTAACC				
		Sequencing	AGATGGTTTTAGATTTTTAGTAT				
GABRB1	cg23231631	Forward	Biotin‐GTTAGAAAGTTAGTAAGGTGGATGGATGAT	0.0320	0.1776	0.9247	0.7471
		Reverse	CCCCATAAAACTCATTAAACACTATATTCC				
		Sequencing	AATATACCTCCCTAATATAAAAATA				
KCNQ2	cg11495604	Forward	GGGTGGGGAGTTAGGTAG	0.0166	0.4154	0.8301	0.4146
		Reverse	Biotin‐CCTCCTCCAACACCAAAACAAAAAACAATA				
		Sequencing	GGGGAGTTAGGTAGG				
CIZ1	cg09976142	Forward	GATTAGTAGTGGAAGAGTTAGGATTTAGAG	0.0359	0.2940	0.6130	0.3190
		Reverse	Biotin‐CCACCCTCCAAACTACCTAT				
		Sequencing	AGAGTTAGGATTTAGAGG				
GNG7	cg19851563	Forward	GGGGGATGATTAAGGTTATGTGAGAG	0.0119	0.1524	0.7304	0.5780
		Reverse	Biotin‐CCCCAAACCAAAATAACAACATTTCCTCA				
		Sequencing	ATGTGAGAGTTTGTAAGT				

* The *p* values shown are nominal (uncorrected) and were generated from an initial discovery‐phase microarray analysis.

#### 2.7.2 Dual Luciferase Reporter Gene Assay

The dual‐luciferase reporter gene assay was conducted to investigate the regulatory role of a specific region within the human GABRB1 gene. The sequences of long and short fragments of GABRB1 (with or without the target region) are detailed in Supporting Information S1; the short fragments lacked the target region, and the sequence in target region was as follows: gctagaaagctagcaaggtggatggatgatgatgatagatagatagatagatagatagatagatagatagatCGatCGatctatctccacatcagggaggcacatcaagccagatgtttaggaacacagtgtttaatgagccttatgggg.

The experimental workflow involved vector construction, gene synthesis, transformation into *Escherichia coli* , plasmid mini‐prep, restriction enzyme digestion and ligation, re‐transformation, plasmid mini‐prep, restriction mapping, sequencing verification and large‐scale plasmid purification. Subsequently, cells were cultured, plated and subjected to transfection experiments. Four transfection groups were established: (1) untransfected control cells (Mock), (2) cells transfected with the empty pGL3‐promoter vector (NC), (3) cells transfected with pGL3‐promoter harbouring the full‐length GABRB1 fragment containing the target region (pGL3‐promoter‐GABRB1‐Long), and (4) cells transfected with pGL3‐promoter carrying the truncated GABRB1 fragment excluding the target region (pGL3‐promoter‐GABRB1‐Short). Then these vectors were transfected into 293T cells using Lipofectamine 2000 transfection reagent, and dual‐luciferase activity was assayed after transfection.

### 2.8 Data Analysis Methods

All statistical analyses were performed using IBM SPSS Statistics 26 (SPSS Inc., Chicago, IL, USA) and GraphPad Prism 8.0 (GraphPad Software Inc La Jolla, CA, USA) and R (version 4.3.1, R Core Team, Vienna, Austria). For the Methylation‐Wide Association Study (MWAS), the identification of DMPs was conducted using R software. For the pyrosequencing, methylation differences between methamphetamine‐dependent individuals and healthy controls were evaluated using linear regression models, adjusting for age as a covariate. For CpG sites where data did not meet the assumptions of parametric tests, appropriate non‐parametric methods were used, also with adjustment for age. The raw *p* values generated from all CpG site comparisons were collectively adjusted using the Benjamini–Hochberg (BH) method to control the false discovery rate (FDR). Additionally, correlation analysis was conducted to explore the relationship between DNA methylation patterns and individual drug use characteristics, such as duration and frequency of drug use. For the statistical analysis of the dual‐luciferase reporter gene assay, one‐way ANOVA was used for normally distributed data, followed by Tukey's post hoc test for multiple comparisons. Kruskal–Wallis test was applied for non‐parametric data, with Dunn's post hoc correction used for multiple comparisons. All data were expressed as the means ± standard deviations (SDs) for normally distributed data, or as median with interquartile range (IQR) for nonnormally distributed data. The *p* < 0.05 was considered indicative of a statistically significant difference.

## 3 Results

### 3.1 Main Findings of Whole Genome Methylation Differences

**FIGURE 1:**
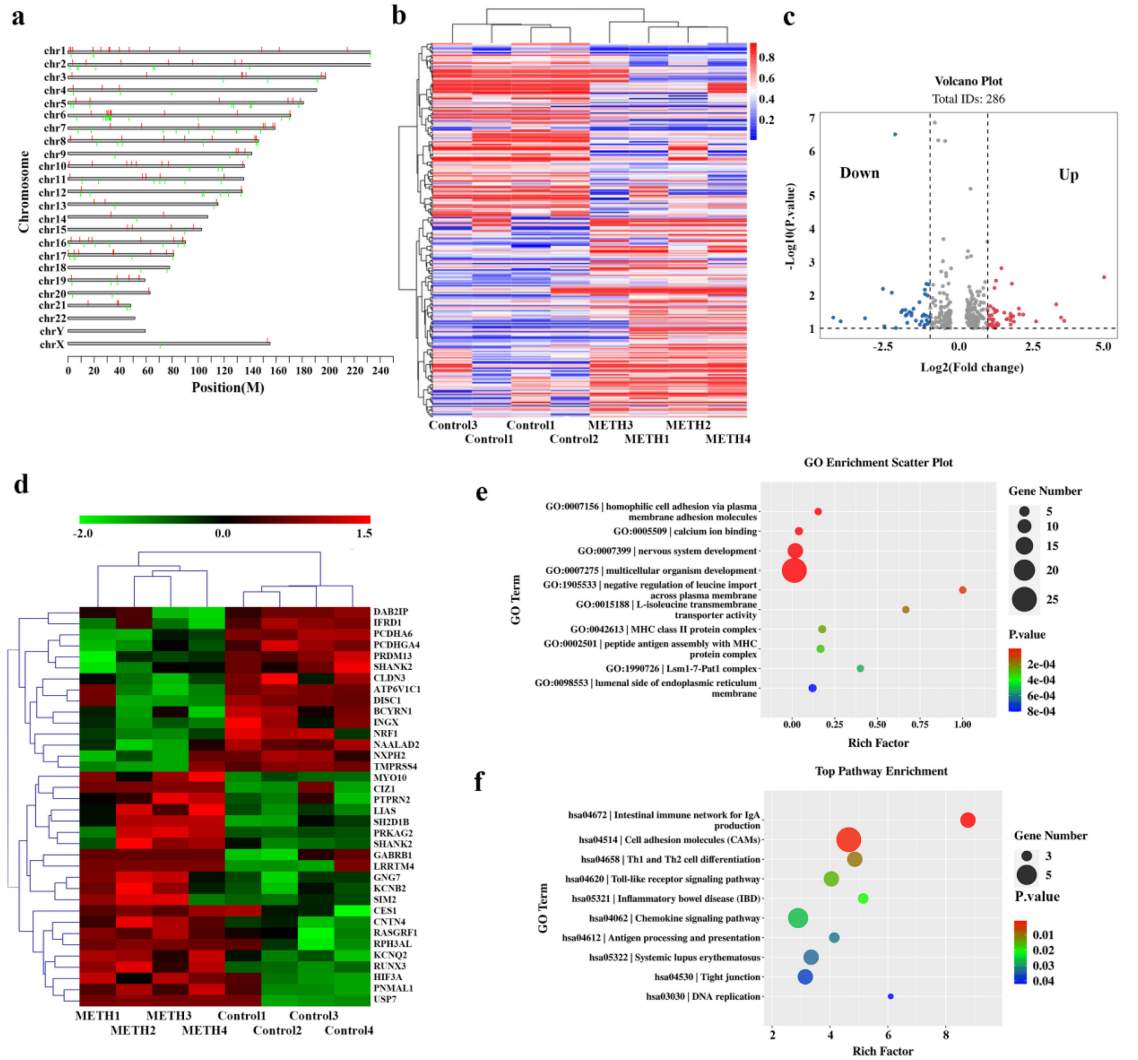
Genome‐wide analysis of differential methylation patterns associated with methamphetamine addiction. (a) Genome‐wide distribution of differentially methylated genes (DMGs). (b) Hierarchical clustering of differentially methylated sites. (c) Volcano plot of DMGs. The *x*‐axis represented log2‐transformed fold change (FC) values, and the y‐axis represented the statistical significance (−log10‐transformed *p* values). (d) Heatmap of DMGs identified through integration with the KARG database. (e) Gene Ontology (GO) enrichment analysis of DMGs. (f) Kyoto Encyclopedia of Genes and Genomes (KEGG) pathway enrichment analysis. The rich factor was calculated as the ratio of the number of DMGs enriched in a specific pathway to the total number of genes associated with that pathway in the background gene set.

Illumina methylation array identified a total of 485 512 methylation sites across the genome. The results of the methylation analysis were displayed in the Figure 1a, which illustrated differential methylation across various chromosomes. The vertical red lines in every chromosome indicated enhanced methylation levels at these specific positions, the green lines represented regions with reduced methylation. Each chromosome displayed a unique pattern of methylation alterations, with varying degrees of both increased and decreased in methylation levels. The chromosome 18 only had regions with significant methylation reduction. There were no sites with significant methylation differences on chromosome 22 and Y.

Upon differential methylation analysis, 133 hypermethylated and 153 hypomethylated sites were discovered, amounting to 286 DMPs. Heatmaps were generated to visualize these genes, as shown in Figure 1b (organizing the DMPs along rows and the samples along the columns, red for high methylation, blue for low methylation).

To simultaneously visualize the statistical significance and magnitude of change for each methylation site, a volcano plot was constructed; the *x*‐axis was the differential methylation effect (log2 fold‐change), and the *y*‐axis was the significance (−log10 (*p* value)) for each methylation site. Colouring or highlighting the points that correspond to the DMPs, and adding horizontal and vertical lines to indicate the significance and fold‐change thresholds, respectively (Figure 1c).

Prioritization of addiction‐relevant genes was achieved by intersecting the DMP‐associated genes with the KARG database. This intersection yielded 15 hypomethylated and 21 hypermethylated genes potentially related to methamphetamine dependence (Figure 1d).

The top 10 enriched GO annotations were shown in Figure 1e, homophilic cell adhesion via plasma membrane adhesion molecules, calcium ion binding, nervous system development, multicellular organism development, negative regulation of leucine import across plasma membrane, L‐isoleucine transmembrane transporter activity, MHC class II protein complex, peptide antigen assembly with MHC protein complex, Lsm1‐7‐Pat1 complex and lumenal side of endoplasmic reticulum membrane. As Figure 1f showed, the KEGG pathway analysis revealed that the intestinal immune network for IgA production, cell adhesion molecules (CAMs), Th1 and Th2 cell differentiation, Toll‐like receptor signalling pathway, inflammatory bowel disease (IBD), chemokine signalling pathway, antigen processing and presentation, systemic lupus erythematosus, tight junction and DNA replication were enriched.

### 3.2 Validation of Candidate Genes by Pyrosequencing

**FIGURE 2:**
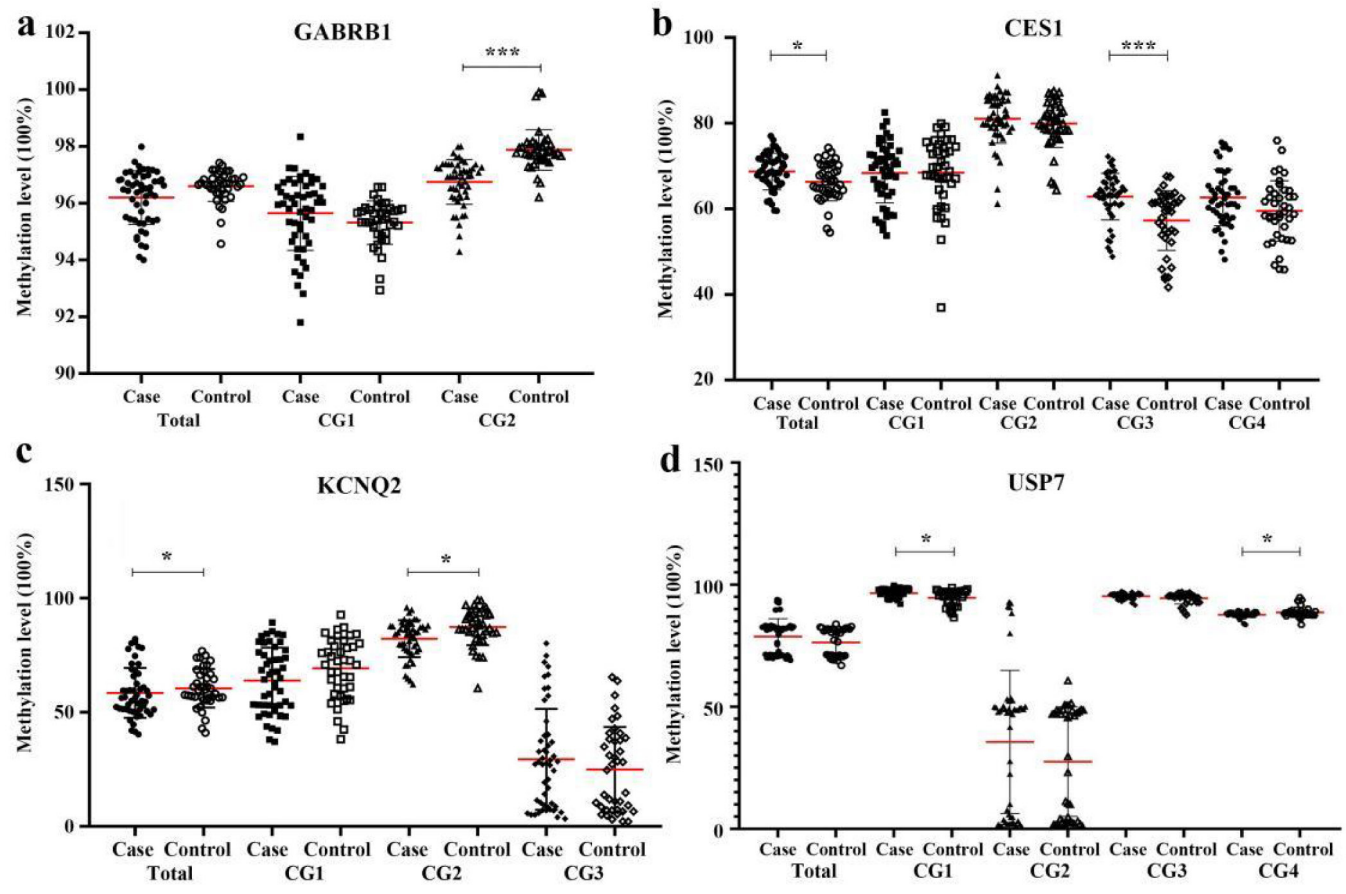
Pyrosequencing validation of methylation levels in five genes associated with methamphetamine addiction. (a) Methylation levels of the GABRB1 gene in methamphetamine‐dependent individuals and controls. (b) Methylation levels of the CES1 gene in methamphetamine‐dependent individuals and controls. (c) Methylation levels of the KCNQ2 gene in methamphetamine‐dependent individuals and controls. (d) Methylation levels of the USP7 gene in methamphetamine‐dependent individuals and controls. Data are presented as mean ± standard deviation. Case group: methamphetamine‐dependent individuals. Statistical significance is indicated as follows: *p ≤ 0.05, ***p ≤ 0.001.

To validate our array‐based findings, eight candidate genes were selected from the 36 DMGs previously identified through integration with the KARG database (Figure 1d). This selection was prioritized based on the magnitude of differential methylation, biological plausibility and previously reported associations with neurological or addiction‐related pathways. After correcting for multiple comparisons using the BH method, several sites were found to be significantly differentially methylated between the case and control groups (all FDR < 0.05). The detailed statistical results are presented in Table 2. Also, as illustrated in Figure 2, significant methylation differences were observed in several genes between methamphetamine‐dependent individuals and healthy controls. Specifically, the GABRB1 gene exhibited a significant reduction in methylation level at CG2 (96.67% ± 0.74% vs. 97.88% ± 0.71%, *t* value = −7.25, FDR = 5.65E−09). In contrast, the CES1 gene showed a significant increase in average methylation levels in methamphetamine‐dependent individuals (68.82% ± 4.04% vs. 65.76% ± 4.37%; *t* value = 3.42, FDR = 0.011), with site‐specific analysis revealing significant hypermethylation at CG3 (median: 63.24% vs. 59.60%; *W* statistic = 1509.5, FDR = 6.30E−04). For the KCNQ2 gene, methylation chip data identified one differential methylation site, cg11495604 (CG2). In this study, significant hypomethylation was found for the KCNQ2 average methylation level in cases (mean: 49.08% ± 6.45 vs. 52.55% ± 5.35%; *t* value = −2.87, FDR = 0.036). Site‐specific analysis revealed a significant decrease in methylation at CG2 in methamphetamine‐dependent individuals (median: 84.57% vs. 87.53%; *W* statistic = 640, FDR = 3.07E−02). Similarly, for the USP7 gene, methylation chip data identified one differential methylation site, cg01891583 (CG2). The CG1 methylation level of USP7 was significantly higher in methamphetamine‐dependent individuals (median: 96.92% vs. 96.02%; *W* statistic = 1335, FDR = 0.039) and a significant decrease at CG4 (median: 87.87% vs. 88.28%; *W* statistic = 672, FDR = 0.039) was demonstrated. In contrast, no statistically significant differences in methylation levels were observed for the ATP6V1C1, CIZ1, GNG7 and LIAS genes between methamphetamine‐dependent individuals and controls (Table 3).

**TABLE 2:** Statistical summary of differential methylation for candidate CpG sites.

Site	Test_Used	Mean (SD)	Mean (SD)	Median [IQR]	Median [IQR]	Statistic value	*p* raw	FDR
		Case (%)	Control (%)	Case (%)	Control (%)			
GABRB1‐CG2	Linear model	96.67 (0.74)	97.88 (0.71)	96.94 [96.45–97.43]	97.80 [97.55–98.05]	−7.25	1.61E−10	**5.65E−09**
CES1‐CG3	Wilcoxon rank‐sum	62.85 (5.46)	57.27 (6.96)	63.24 [60.71–65.77]	59.60 [55.83–63.37]	1509.50	3.58E−5	**6.30E−04**
CES1‐Means	Linear model	68.82 (4.04)	65.76 (4.34)	69.47 [66.62–72.33]	66.38 [63.70–69.06]	3.42	9.52E−04	**1.11E−02**
KCNQ2‐CG2	Wilcoxon rank‐sum	82.26 (8.08)	87.38 (7.96)	84.57 [79.57–89.58]	87.53 [83.27–91.78]	640.00	3.51E−03	**3.07E−02**
KCNQ2‐Means	Linear model	49.08 (6.49)	52.55 (5.35)	48.73 [42.99–54.46]	53.62 [50.01–57.23]	−2.87	5.15E−03	**3.61E−02**
USP7‐CG1	Wilcoxon rank‐sum	96.58 (1.70)	94.65 (3.27)	96.92 [95.44–98.40]	96.02 [93.70–98.34]	1335.00	6.60E−03	**3.85E−02**
USP7‐CG4	Wilcoxon rank‐sum	87.71 (1.12)	88.73 (2.13)	87.87 [87.40–88.33]	88.28 [87.63–88.94]	672.00	7.83E−03	**3.91E−02**

*Note:* A finding was considered statistically significant if the resulting FDR was less than 0.05.

Abbreviations: FDR, false discovery rate; IQR, interquartile range; SD, standard deviation.

**TABLE 3:** Statistical summary of nondifferentially methylated CpG sites.

Site	Mean (SD)	Mean (SD)	Median [IQR]	Median [IQR]	Statistic Value	*p* raw	FDR
	Case (%)	Control (%)	Case (%)	Control (%)			
GABRB1‐CG1	95.61 (1.32)	95.32 (0.77)	95.97 [95.09–96.84]	95.42 [95.01–95.84]	1262	0.034	0.092
GABRB1‐MEANS	96.17 (0.95)	96.60 (0.54)	96.45 [95.75–97.16]	96.68 [96.45–96.90]	750.5	0.043	0.101
CES1‐CG1	68.39 (6.98)	68.48 (8.44)	69.73 [65.46–74.00]	69.74 [65.35–74.13]	950	0.688	0.83
CES1‐CG2	81.07 (5.73)	79.91 (5.57)	81.22 [78.06–84.38]	80.42 [77.69–83.15]	1136.5	0.269	0.472
CES1‐CG4	62.63 (6.57)	59.54 (7.04)	62.03 [58.13–65.93]	59.05 [54.86–63.23]	2.14	0.036	0.092
ATP6V1C1‐CG1	28.36 (7.56)	32.27 (6.55)	28.72 [23.81–33.63]	31.35 [27.02–35.68]	−2.51	0.014	0.062
ATP6V1C1‐CG2	51.08 (9.93)	55.95 (9.04)	52.69 [45.49–59.89]	55.10 [48.28–61.92]	−2.331	0.022	0.077
ATP6V1C1‐CG3	38.71 (8.00)	42.79 (9.58)	39.80 [34.42–45.19]	41.94 [35.71–48.16]	−2.181	0.03	0.092
ATP6V1C1‐MEANS	39.38 (8.14)	43.67 (7.95)	40.75 [35.56–45.95]	41.81 [36.12–47.50]	−2.44	0.017	0.066
KCNQ2‐CG1	63.97 (14.36)	69.28 (13.25)	64.69 [53.23–76.15]	71.69 [62.00–81.39]	779	0.073	0.161
KCNQ2‐CG3	29.44 (22.13)	24.94 (18.66)	27.49 [12.34–42.65]	25.97 [10.42–41.52]	1083	0.503	0.733
USP7‐CG2	35.66 (29.28)	27.55 (22.45)	47.89 [24.62–71.16]	37.64 [14.86–60.43]	1196	0.112	0.231
USP7‐CG3	95.31 (1.08)	94.45 (2.34)	95.52 [94.98–96.05]	95.41 [94.74–96.09]	1156	0.207	0.381
USP7‐MEANS	78.82 (7.31)	76.35 (5.69)	81.47 [75.72–87.21]	78.40 [72.93–83.87]	1257.5	0.037	0.092
CIZ1‐CG1	39.42 (17.92)	41.71 (18.54)	41.11 [34.48–47.73]	41.36 [31.78–50.94]	965	0.78	0.88
CIZ1‐CG2	42.66 (17.77)	45.25 (19.11)	44.89 [38.64–51.14]	46.24 [35.22–57.26]	928	0.562	0.786
CIZ1‐CG3	34.30 (15.55)	37.37 (15.51)	35.75 [26.86–44.63]	36.58 [28.04–45.11]	896.5	0.403	0.613
CIZ1‐CG4	39.22 (16.31)	42.34 (17.21)	42.63 [33.26–52.00]	42.13 [32.54–51.72]	896.5	0.403	0.613
CIZ1‐CG5	37.92 (14.44)	40.00 (15.75)	40.38 [32.87–47.90]	38.83 [30.64–47.01]	949	0.682	0.83
CIZ1‐MEANS	31.32 (13.33)	33.53 (13.95)	32.91 [27.09–38.72]	32.94 [25.43–40.45]	935.5	0.603	0.812
GNG7‐CG1	46.15 (10.94)	45.94 (11.27)	45.09 [38.01–52.17]	44.61 [36.58–52.63]	0.14	0.892	0.917
GNG7‐CG2	49.84 (10.27)	48.90 (11.01)	47.55 [40.63–54.48]	47.80 [40.54–55.07]	0.44	0.664	0.83
GNG7‐CG3	47.91 (10.89)	48.14 (10.88)	46.75 [40.42–53.07]	46.75 [39.66–53.83]	−0.10	0.917	0.917
GNG7‐MEANS	47.97 (10.30)	47.66 (10.73)	46.80 [40.54–53.07]	45.05 [38.24–51.85]	0.16	0.875	0.917
LIAS‐CG1	17.74 (6.89)	17.41 (5.13)	17.37 [12.59–22.15]	17.08 [12.81–21.34]	0.29	0.769	0.88
LIAS‐CG2	33.11 (15.89)	33.00 (13.46)	35.30 [23.21–47.39]	39.58 [27.49–51.67]	1023	0.855	0.917
LIAS‐CG3	29.80 (12.86)	28.01 (10.06)	34.55 [23.66–45.45]	33.22 [24.06–42.37]	1164	0.184	0.358
LIAS‐MEANS	26.88 (11.25)	26.14 (8.64)	31.23 [22.65–39.80]	30.24 [23.63–36.86]	1111.5	0.367	0.612

### 3.3 Diagnostic Potential and Functional Characterization

**FIGURE 3:**
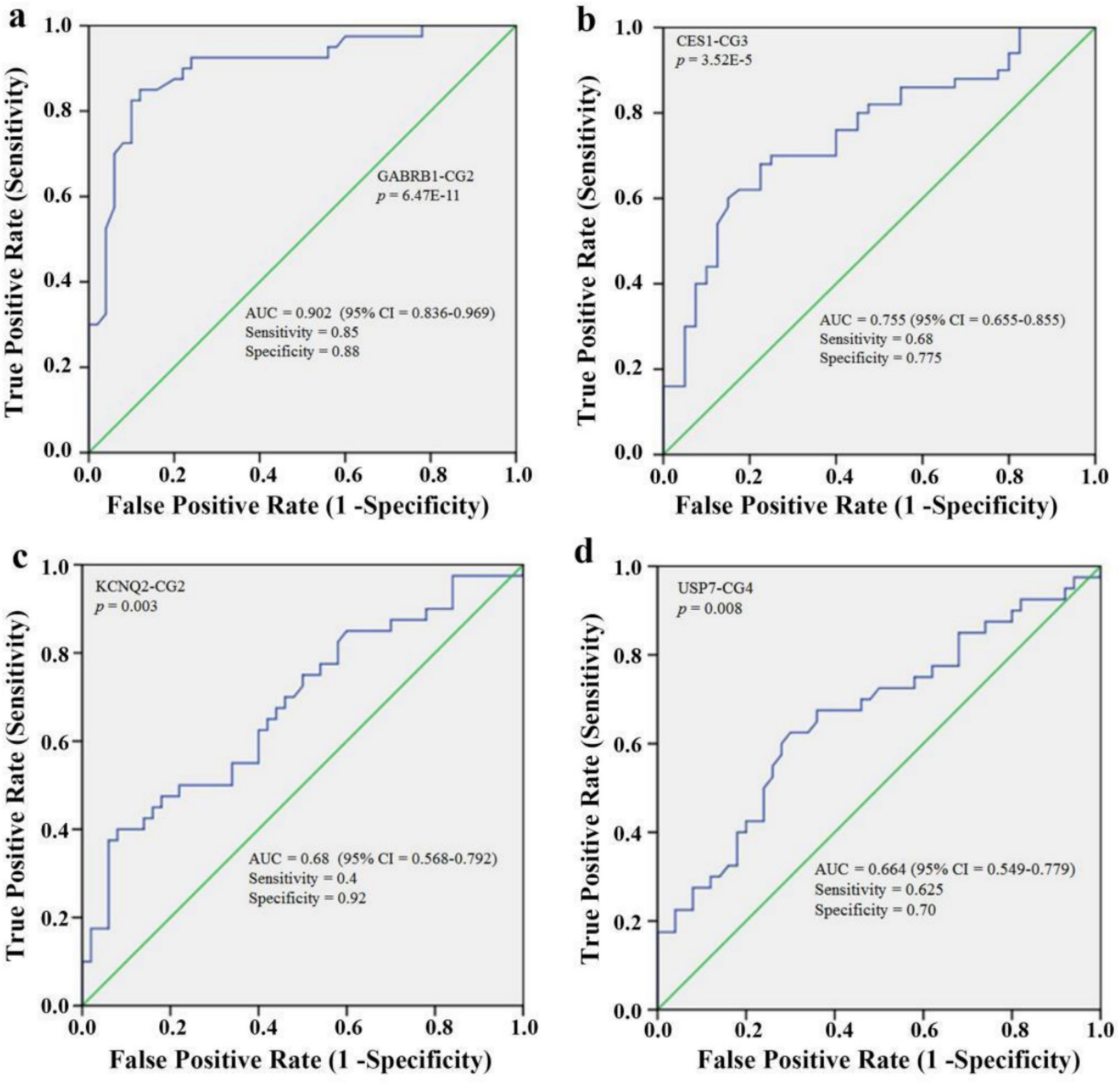
ROC curves for differentiation of four genes (GABRB1, CES1, KCNQ2, USP7) between methamphetamine‐dependent individuals and controls. (a) ROC curve for GABRB1 CG2 site. (b) ROC curve for CES1 CG3 site. (c) ROC curve for KCNQ2 CG2 site. (d) ROC curve for USP7 CG4 site.

To evaluate the diagnostic potential of methylation markers for methamphetamine dependence, ROC curve analyses were performed on the identified genes. These analyses revealed several key methylation sites with significant diagnostic value, suggesting their potential as biomarkers for this condition. As illustrated in Figure 3, the methylation level of CG2 within the GABRB1 gene exhibited remarkable diagnostic significance for methamphetamine dependence (*p* = 6.47E−11), with an area under the ROC curve (AUC) of 0.902 (95% CI: 0.836–0.969), a sensitivity of 0.85 and a specificity of 0.88. Similarly, the methylation level of CG3 in the CES1 gene also demonstrated significant diagnostic value (*p* = 3.52E−5), achieving an AUC of 0.755 (95% CI: 0.655–0.855), with a sensitivity of 0.68 and a specificity of 0.775.

Additionally, the methylation level of CG2 in the KCNQ2 gene was significantly associated with methamphetamine dependence (*p* = 0.003), yielding an AUC of 0.68 (95% CI: 0.568–0.792), a sensitivity of 0.40 and a specificity of 0.92. Furthermore, the methylation level of CG4 in the USP7 gene demonstrated significant diagnostic relevance (*p* = 0.008), with an AUC of 0.664 (95% CI: 0.549–0.779), a sensitivity of 0.625 and a specificity of 0.70. In contrast, ROC curve analysis indicated no diagnostic significance for methylation in the ATP6V1C1, CIZ1, GNG7 and LIAS genes concerning methamphetamine dependence.

**FIGURE 4:**
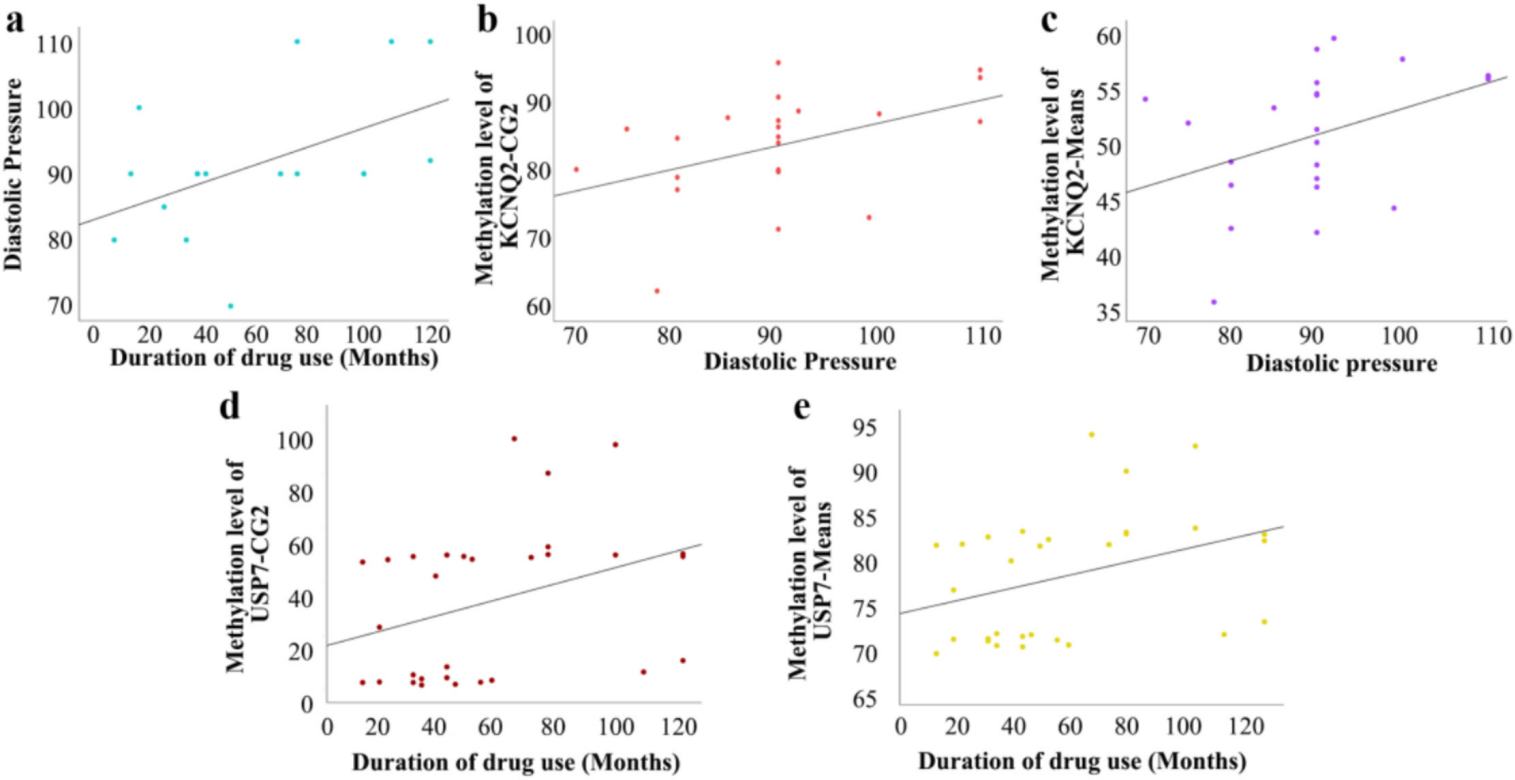
Correlation analysis of methylation levels with biochemical markers and drug use characteristics in methamphetamine‐dependent individuals. (a) Correlation between diastolic blood pressure and the duration of methamphetamine use. (b) Correlation between diastolic blood pressure and the methylation level of CG2 in the KCNQ2 gene. (c) Correlation between diastolic blood pressure and the overall methylation levels of the KCNQ2 gene. (d) Correlation between the duration of drug use and the methylation level at CG2 in the USP7 gene. (e) Correlation between the duration of drug use and the overall methylation levels of the USP7 gene.

Furthermore, we conducted a correlation analysis of methylation levels in relation to biochemical markers in individuals with substance use disorders, as well as their frequency and quantity of drug use. The results indicated a positive correlation between diastolic pressure values and the duration of methamphetamine use (*p* = 0.019, *r* = 0.560) (Figure 4a). Additionally, diastolic pressure was found to be significantly associated with the methylation level of CG2 in the KCNQ2 gene (*p* = 0.024, *r* = 0.470) (Figure 4b) and the overall methylation levels (*p* = 0.014, *r* = 0.530) (Figure 4c). Methylation level at CG2 (*p* = 0.003, *r* = 0.524) (Figure 4d) and the overall methylation levels (*p* = 0.008, *r* = 0.476) (Figure 4e) in the USP7 gene were positively correlated with the duration of drug use.

**FIGURE 5:**
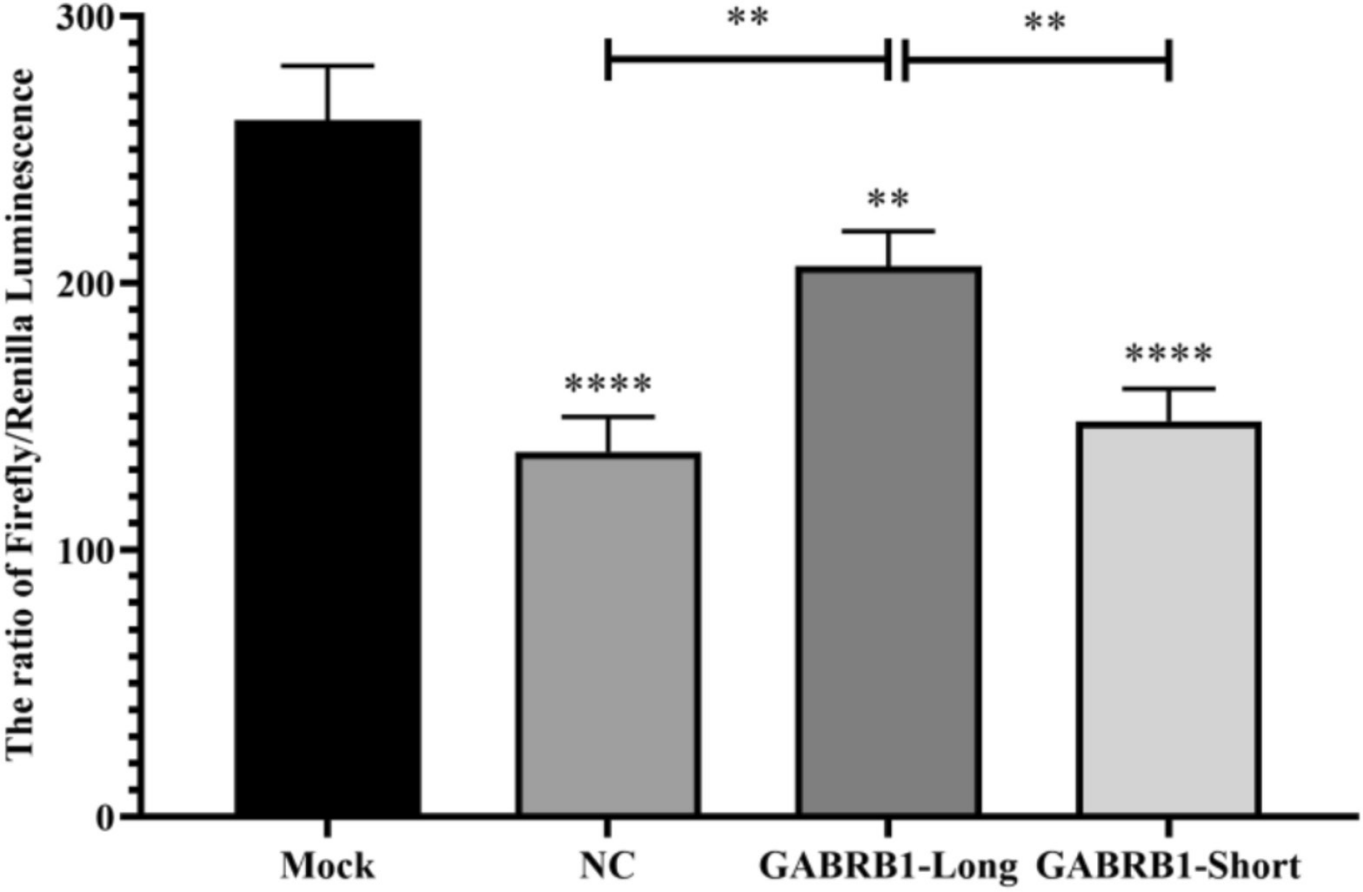
Luciferase validation results after transfection of GABRB1‐Long/Short fragments. Mock: untransfected control cells, NC: cells transfected with the empty pGL3‐promoter vector, GABRB1‐Long: cells transfected with pGL3‐promoter harbouring the full‐length GABRB1 fragment containing the target region, GABRB1‐Short: cells transfected with pGL3‐promoter carrying the truncated GABRB1 fragment excluding the target region. **p < 0.01, ***p < 0.0001.

The dual‐luciferase reporter assay demonstrated significantly enhanced luciferase activity in the pGL3‐promoter‐GABRB1‐Long group compared with controls (136.65 ± 13.11 vs. 206.1 ± 13.36, *p* < 0.01), while there was no significant change observed in the pGL3‐promoter‐GABRB1‐Short fragment group compared with the NC group (136.65 ± 13.11 vs. 148.17 ± 12.16, *p* < 0.01). Compared with Mock (261 ± 20.38, *p* < 0.01), all the luciferase activities in the other three groups decreased (*p* < 0.01) (Figure 5). This may indicate that the deleted region was likely crucial for this enhancement effect, providing evidence that our tested GABRB1 fragment might act as an enhancer, potentially playing a regulatory role in gene expression.

## 4 Discussion

This study provides a comprehensive, genome‐wide analysis of DNA methylation in methamphetamine‐dependent individuals, which encompassed the identification of 485 512 methylation sites and the categorization of 286 DMPs, providing a novel perspective on the role of methylation in addiction. Our findings not only identify novel biological pathways and candidate genes but also validate a highly accurate biomarker with immediate translational potential. This was confirmed by the finding that following chronic methamphetamine exposure, there was a decrease in 5′‐methylcytosine and 5′‐hydroxymethylcytosine levels at the promoter region of these genes [15].

The GO analysis of DMGs revealed notable enriched annotations, including homophilic cell adhesion, calcium ion binding and nervous system development, highlighting the importance of cell communication, metabolic regulation and neurodevelopmental processes. Additionally, the DMGs were involved in immune responses, particularly through pathways related to MHC class II protein complexes and antigen assembly, suggesting implications for susceptibility to infections and autoimmune diseases. The KEGG pathway analysis further linked the DMGs to critical processes such as the intestinal immune network for IgA production, Th1 and Th2 cell differentiation, and Toll‐like receptor signalling, which suggests that chronic methamphetamine use compromises gut barrier integrity, potentially initiating systemic inflammation through the gut‐brain axis [16]. Furthermore, the influence on Th1 and Th2 cell differentiation indicates that this acute response transitions into a chronic, proinflammatory state, altering the body's adaptive immune landscape. Collectively, these findings suggest that methamphetamine may orchestrate a systemic, self‐perpetuating inflammatory cycle that begins in the gut and ultimately fuels the neuroinflammation known to drive the progression and severity of addiction. These findings are consistent with emerging evidence implicating neuroimmune interactions in addiction, which suggested that methamphetamine induces blood–brain barrier damage through multiple mechanisms, particularly by triggering inflammatory responses in both the peripheral and central nervous systems, which further exacerbates blood–brain barrier dysfunction [17, 18]. Prolonged methamphetamine exposure triggers systemic inflammation, characterized by a significant increase in inflammatory mediators [19].

The validation of eight genes through pyrosequencing in our study highlights significant alterations in DNA methylation patterns associated with methamphetamine dependence. Notably, the observed hypomethylation of GABRB1, and KCNQ2, along with the hypermethylation of CES1 and USP7, may underlie the pathophysiology of methamphetamine dependence.

The GABRB1 gene encodes one of the GABA_A receptors, which is critical for inhibitory neurotransmission [20]. Here GABRB1 exhibited a significant decrease in average methylation levels, especially in the CG2 site of GABRB1 in methamphetamine‐dependent individuals. The high AUC values indicated excellent diagnostic performance; besides, the target fragments demonstrated associations with enhanced gene transcription. Thus, the observed hypomethylation of GABRB1 may suggest a potential increase in transcriptional activity, likely increasing GABAergic signaling to restore neuronal homeostasis, potentially representing a neuroadaptive response to the chronic stress and neurotransmitter imbalances associated with methamphetamine use [21, 22, 23]. Preclinical and clinical investigations focusing on GABA system‐targeting medications for addiction treatment have emerged as an active research area [24, 25]. For instance, studies demonstrated the potential of GABA receptor agonists in reducing alcohol consumption [26], suggesting that pharmacological agents such as Baclofen, GABA analogues like Gabapentin and topiramate might offer promising therapeutic approaches for alcohol and drug abuse treatment [20, 27]. The high AUC value (area under the curve) of GABRB1 methylation level indicated that it has excellent diagnostic performance and may be used as a biomarker for methamphetamine dependence. By detecting the methylation status of GABRB1, it may help to identify methamphetamine‐dependent individuals early and provide a basis for personalized treatment.

Critically, methylation at the KCNQ2 locus was also positively correlated with diastolic blood pressure, providing a direct mechanistic link between a specific epigenetic mark and the known cardiovascular toxicity of methamphetamine. The KCNQ2 gene, which is part of the Kv7.2 family of voltage‐gated potassium channels, is known to play crucial roles in the nervous system, heart, muscle and epithelia. Previous studies have demonstrated the importance of KCNQ2 in regulating neuronal hyperexcitability [28]. The therapeutic potential of targeting Kv7 channels, already demonstrated in epilepsy [29, 30, 31], stroke‐induced injury [32] and alcohol use disorders [31, 33, 34] may therefore extend to the treatment of methamphetamine dependence.

In contrast, CES1 and USP7 exhibited hypermethylation in methamphetamine‐dependent individuals. Besides CES1 showed significant diagnostic value. CES1 encodes carboxylesterase 1 and is the most abundant hepatic drug‐metabolizing enzyme in the liver [35]. Loss‐of‐function variants of CES1 have been shown to have significant effects on the metabolism and elimination of a variety of drugs, including amphetamine‐type psychostimulants (such as methylphenidate) [36]. This strongly suggests that chronic use may epigenetically suppress the body's own ability to clear methamphetamine. Such a mechanism would create a vicious cycle: chronic exposure diminishes metabolic efficiency, thereby prolonging the drug's half‐life and tissue exposure, which in turn exacerbates its neurotoxicity and systemic damage with each subsequent use.

Perhaps our most significant finding is that methylation levels of the USP7 gene are positively correlated with the duration of drug use. USP7 is predominantly involved in the response to DNA damage and stress [37]. The progressive methylation of USP7 may function as an ‘epigenetic clock’ or ‘molecular scar’, quantifying the cumulative cellular damage inflicted by the addictive process. This provides a tangible molecular basis for the chronicity and escalating severity of addiction. Previous studies have found that children carrying USP7 mutations and deletions may be associated with neurodevelopmental disorders such as autism spectrum disorders, intellectual disabilities and speech/movement disorders [38].

A previous study had shown that pharmacological inhibition of USP7 prevents cocaine‐induced behavioural sensitization, suggesting USP7 may serve as a marker of risk factors [39]. Taken together, the epigenetic regulation of CES1 and USP7 shifts the research focus from a model of simple neurochemical imbalance to one of systemic, progressive toxicity. These genes are not only high‐potential biomarkers for assessing disease severity and metabolic risk but also represent novel therapeutic targets. For instance, modulating USP7 activity could become a pioneering strategy to mitigate the long‐term, multi‐organ damage that renders recovery from addiction so challenging.

The positive correlation between diastolic blood pressure and the duration of methamphetamine use raises significant concerns regarding cardiovascular health among users, underscoring the profound and progressive cardiovascular toxicity of chronic methamphetamine exposure. This finding aligns with previous research that has shown cardiovascular measures, including blood pressure, to be elevated following methamphetamine exposure [40]. Emerging evidence now suggests that the chronic, low‐grade systemic inflammation induced by methamphetamine—a state strongly supported by our own epigenetic data, which implicates the intestinal immune network and Toll‐like receptor signaling—is a primary driver of this vascular damage. This pro‐inflammatory environment directly injures the vascular endothelium, resulting in reduced bioavailability of nitric oxide (NO), impaired vasodilation and increased expression of adhesion molecules that promote atherosclerosis [41]. Therefore, the elevated blood pressure we observe may be a clinical manifestation of this underlying inflammatory vascular pathology, which worsens with cumulative exposure. This perspective reframes the cardiovascular complications of methamphetamine use not as an isolated side effect, but as a direct consequence of the same systemic inflammatory cycle that drives its neurotoxicity. Notably, previous studies showed that pharmacological interventions with enhanced brain acetylcholine can attenuate methamphetamine‐induced increases in diastolic blood pressure [42], highlighting potential therapeutic avenues for addressing cardiovascular complications associated with methamphetamine use. Future research should investigate the potential of agents with anti‐inflammatory or endothelial‐protective properties—such as statins, ACE inhibitors or novel immunomodulatory drugs—as adjunct treatments in methamphetamine dependence. Such interventions would not only aim to manage hypertension but could potentially mitigate the broader systemic and neurological damage, highlighting the urgent need for integrated care models that address both the psychiatric and cardiovascular health of individuals with methamphetamine use disorder.

While this study provides valuable insights, it is not without limitations. Limitations of this study include the relatively small sample size, which may have restricted the generalizability of our findings. Additionally, the study exclusively enrolled male participants, excluding females, which further limits the generalizability of the results across genders and may overlook gender‐specific patterns in methylation changes related to addiction. Moreover, while stringent age and sex matching were employed (though only within the male cohort), DNAm‐based cell composition analysis was not performed, which represents a potential source of cellular heterogeneity influencing methylation patterns in peripheral blood samples. Furthermore, the validation of candidate genes via pyrosequencing was conducted on the same cohort used for the initial MWAS. While this approach served to confirm initial methylation differences, it inherently carries a risk of cohort‐dependent overfitting, potentially leading to an overestimation of the observed associations and predictive values (e.g., AUC). Future research should focus on further validating the identified DMPs and their associated genes in larger and more diverse populations (including both males and females), incorporating DNAm‐based cell composition analysis. And crucially, utilizing independent cohorts to validate candidate genes and assess their predictive performance (e.g., AUC values) to ensure generalizability. Furthermore, the cross‐sectional nature of the study limits our ability to infer causality between methylation changes and addiction. Future research should address these limitations by incorporating larger, more diverse populations and longitudinal designs to better understand the temporal dynamics of methylation changes in relation to substance use. What is more, the exact mechanisms behind the observed methylation changes remain unclear and warrant further investigation.

In conclusion, our study identifies significant methylation alterations in GABRB1, ATP6V1C1, KCNQ2, CES1 and USP7 in methamphetamine‐dependent individuals, highlighting their potential as diagnostic biomarkers and therapeutic targets. The functional implications of these changes, particularly in neural signalling, drug metabolism and neuroimmune interactions, provide valuable insights into the pathophysiology of methamphetamine addiction. Future research should focus on validating these findings and exploring their clinical applications to improve the diagnosis and treatment of this debilitating disorder.

## Author Contributions

Qingxiao Hong designed the study, performed data analysis and wrote the initial draft. Shanshan Chen and Zhongze Lou conducted the pyrosequencing validation and biochemical marker analysis. Weisheng Chen, Han Du, Longhui Li, Xiaohu Xie and Wenjin Xu participated in sample collection and clinical data acquisition. Wenhua Zhou and Huifen Liu, as corresponding authors, supervised the entire project, provided oversight and mentorship beyond the core team and revised the manuscript. All authors reviewed and approved the final version of the manuscript.

## Funding

This work is supported by the Natural Science Foundation of China (Grant No. 82471516), the Ningbo Key Research and Development Program (Grant No. 2024Z231), the Natural Science Foundation of Zhejiang Province (Grant No. LQ24H090002), the Medical Health Science and Technology Project of Zhejiang Province (Grant No. 2024KY348, 2023KY1128, 2023KY296), the Zhejiang Medical & Health Leading Academic Discipline Project (Grant No. 00‐F06), the Innovation Project of Distinguished Medical Team in Ningbo (Grant No. 2022030410) and the Natural Science Foundation of Ningbo (Grant No. 2022J270, 2022J207).

## Ethics Statement

The study protocol was reviewed and approved by the Ethics Committee of Ningbo Addiction Research and Treatment Center (Approval No. 201701, China).

## Consent

All participants in this study voluntarily provided written informed consent prior to their inclusion. Written informed consent forms were obtained from every participant.

## Conflicts of Interest

The authors declare no conflicts of interest.

## Supporting information


**Data S1:** Supporting Information.

**Data S2:** Supporting Information.

## Data Availability

Our Illumina microarray data have been successfully submitted to the GEO database and will be publicly released on December 31, 2026. The accession number for citing these data is GSE293262. The corresponding SRA records will also be accessible via the following link after the release date: https://www.ncbi.nlm.nih.gov/geo/query/acc.cgi?acc=GSE293262.
