# Supplementary material for: Epigenetic Landscapes of Methamphetamine Addiction: Unravelling the Diagnostic Potential of Gene Methylation
Source: Addict Biol. 2025 Dec 10;30(12):e70108. doi: 10.1111/adb.70108 (PMC12690404; doi:10.1111/adb.70108)
Supplement: Supplementary file 1 — Data S1: Supporting Information. [file ADB-30-e70108-s001.docx]

**Supplementary Materials:**

**GABRB1-Long:**

gctagaaagctagcaaggtggatggatgatgatgatagatagatagatagatagatagatagatagatagatCGatCGatctatctccacatcagggaggcacatcaagccagatgtttaggaacacagtgtttaatgagccttatggggtatcattagtaacaggttaatgaaaacaggcaaaggtcctgcaacttgtgtcCGagcctgttctttttgccatcagGTCGACGCCCACGGTAACATTCTCCTCAGCACCCTGGAAATCCGGAATGAGACGAGTGGCTCGGAAGTGCTCACGAGCGTGAGCGACCCCAAGGCCACCATGTACTCCTATGACAGCGCCAGCATCCAGTACCGCAAGCCCCTGAGCAGCCGCGAGGCCTACGGGCGCGCCCTGGACCGGCACGGGGTACCCAGCAAGGGGCGCATCCGCAGGCGTGCCTCCCAGCTCAAAGTCAAGATCCCCGACTTGACTGATGTGAATTCCATAGACAAGTGGTCCCGAATGTTTTTCCCCATCACCTTTTCTCTTTTTAATGTCGTCTATTGGCTTTACTATGTACACTGAGGTCTGTTCTAATGGTTCCATTTAGACTACTTTCCTCTTCTATTGTTTTTTAACCTTACAGGTCCCCAACAGCGATACTGCTGTTTCTCGAGGTAAGAGATTCAGCCATCCAATTGGTTTTAGGTCTTGCATATCAGTTTTATTACTGCACCATGTTTACTTCAAAAAGACAAAACAAAAAAAAAATTATTTTTCCAGTCTACCGTGGTCCAGGTTATCAGCTCTTTAAGAGCTCTATTAATTGCCATGTTTACAAACAAACACAAAGAGAGAAGTTAGACAGGTAGATCTTTAGCAGTCTTTTCTAGTTTCCCTGGATTTCACTGATTTATTTTTTAGGGAAAATGAAAAGAGGACCTTGCTGTCCGCCTGCACTGCTTCCTGGTAAACTATAACAAACTTATGCTGCCAAAAAAAAAAAAAAAA

**GABRB1-Short:**

tatcattagtaacaggttaatgaaaacaggcaaaggtcctgcaacttgtgtcCGagcctgttctttttgccatcagGTCGACGCCCACGGTAACATTCTCCTCAGCACCCTGGAAATCCGGAATGAGACGAGTGGCTCGGAAGTGCTCACGAGCGTGAGCGACCCCAAGGCCACCATGTACTCCTATGACAGCGCCAGCATCCAGTACCGCAAGCCCCTGAGCAGCCGCGAGGCCTACGGGCGCGCCCTGGACCGGCACGGGGTACCCAGCAAGGGGCGCATCCGCAGGCGTGCCTCCCAGCTCAAAGTCAAGATCCCCGACTTGACTGATGTGAATTCCATAGACAAGTGGTCCCGAATGTTTTTCCCCATCACCTTTTCTCTTTTTAATGTCGTCTATTGGCTTTACTATGTACACTGAGGTCTGTTCTAATGGTTCCATTTAGACTACTTTCCTCTTCTATTGTTTTTTAACCTTACAGGTCCCCAACAGCGATACTGCTGTTTCTCGAGGTAAGAGATTCAGCCATCCAATTGGTTTTAGGTCTTGCATATCAGTTTTATTACTGCACCATGTTTACTTCAAAAAGACAAAACAAAAAAAAAATTATTTTTCCAGTCTACCGTGGTCCAGGTTATCAGCTCTTTAAGAGCTCTATTAATTGCCATGTTTACAAACAAACACAAAGAGAGAAGTTAGACAGGTAGATCTTTAGCAGTCTTTTCTAGTTTCCCTGGATTTCACTGATTTATTTTTTAGGGAAAATGAAAAGAGGACCTTGCTGTCCGCCTGCACTGCTTCCTGGTAAACTATAACAAACTTATGCTGCCAAAAAAAAAAAAAAAA
